# Supplementary figures and images for: Using a mobile nanopore sequencing lab for end-to-end genomic surveillance of Plasmodium falciparum: A feasibility study
Source: PLOS Glob Public Health. 2024 Feb 1;4(2):e0002743. doi: 10.1371/journal.pgph.0002743 (PMC10833559; doi:10.1371/journal.pgph.0002743)

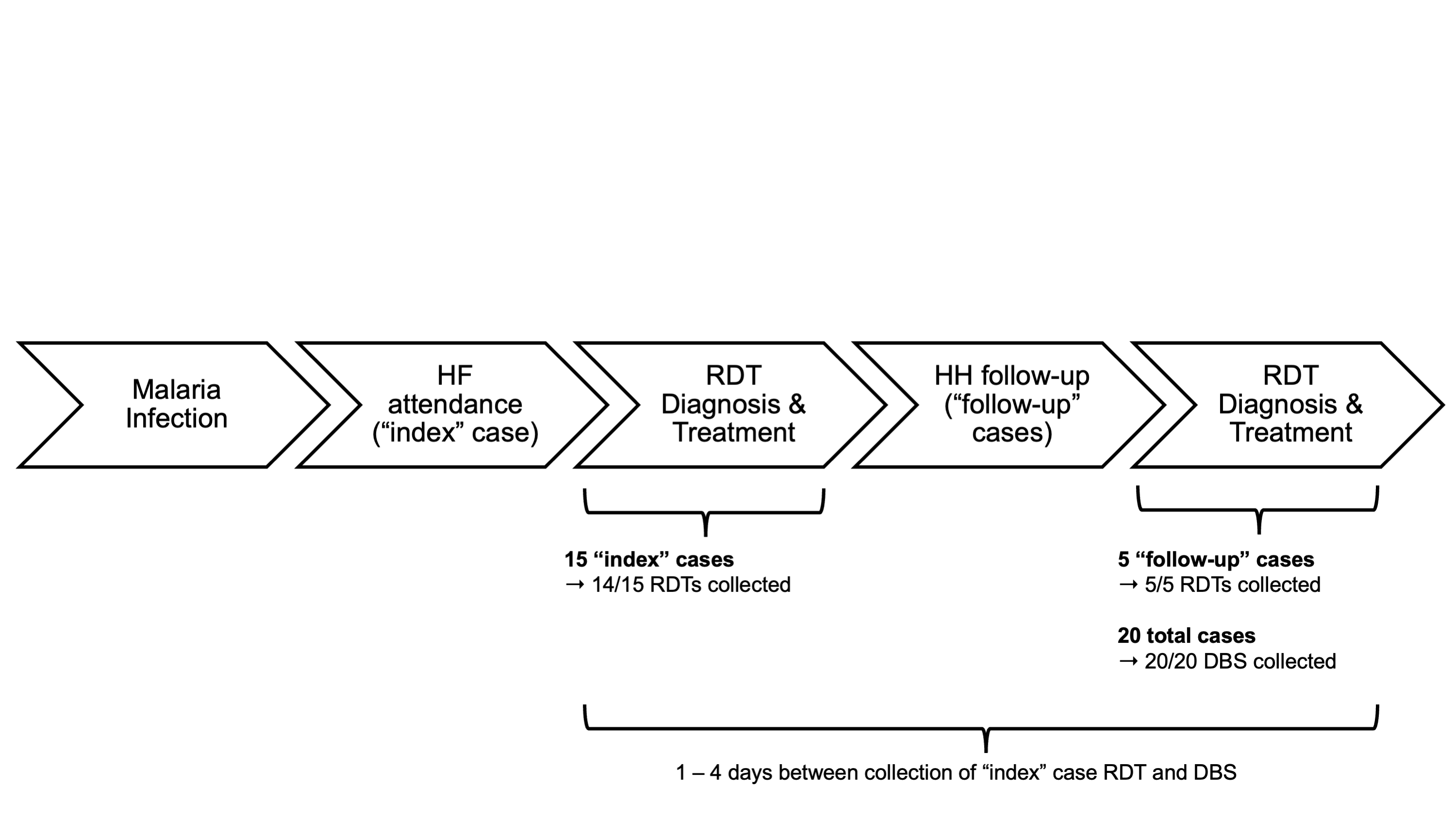

Supplement: S1 Fig — Illustrated from initial diagnosis to reactive response at a malaria patient’s household. Collection of RDT and DBS samples from both, “index” cases and “follow-up” cases are indicated. A total of 20/20 DBS and 19/20 RDTs were available. DBS = dried blood spot; HF = health facility; HH = household; RDT = rapid diagnostic test. (TIFF) [file pgph.0002743.s002.tiff]

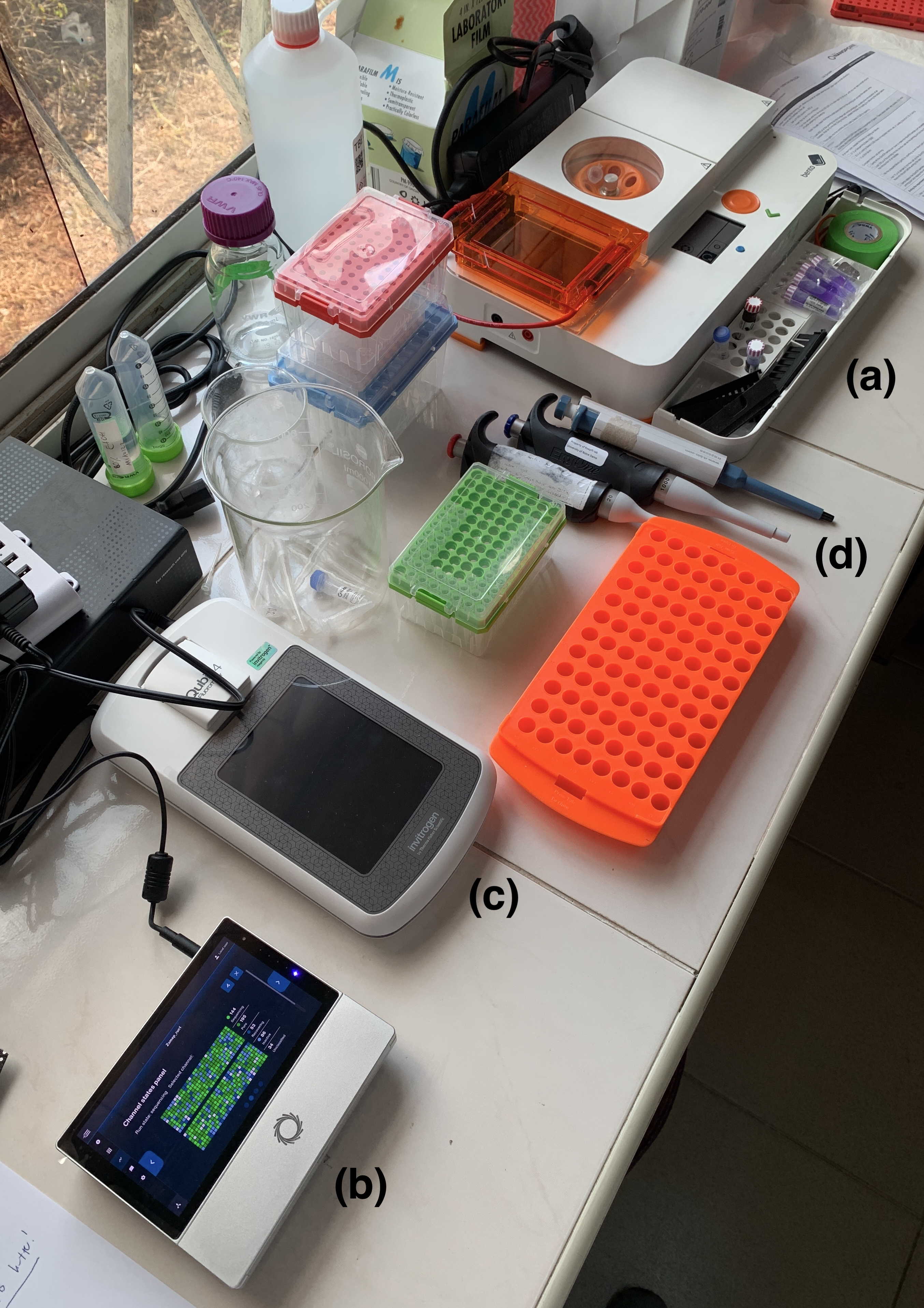

Supplement: S2 Fig — (a) Bento Lab (Bento Bioworks Ltd.) that includes a 32-well PCR thermal cycler, microcentrifuge, and gel electrophoresis apparatus with LED transilluminator. (b) minION Mk1C sequencer (ONT) with fully integrated computer and a screen. (c) Qubit (ThermoFisher) that is needed for DNA quantification during sequencing library preparation. (d) standard laboratory pipettes. Other equipment (not shown here) included a heat block, a mini vortex, and an external SSD drive (SanDisk Extreme Portable SSD, 1TB). For a detailed list see S5 Table. (TIFF) [file pgph.0002743.s003.tiff]

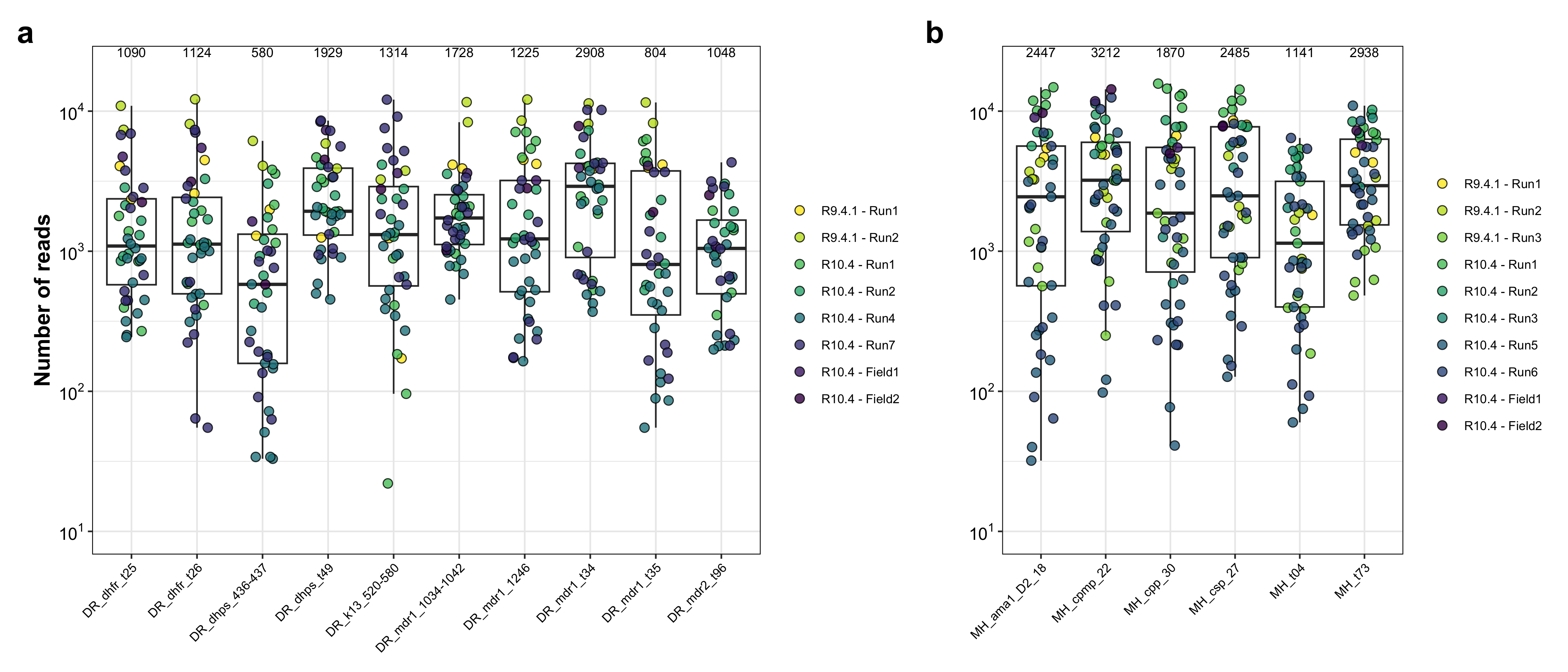

Supplement: S3 Fig — Coverage per marker in control samples for (a) drug resistance panel and (b) microhaplotype panel. y-axis shows number of reads (log10) covering each amplicon target per sample for each of the MinION runs, including control samples from the two MinION runs in the field in Zanzibar. Median coverage for all amplicons is indicated on top of the graph. Note, archived field DBS samples and low-density DBS and RDT control samples (<250 parasites/μL) were excluded. (TIFF) [file pgph.0002743.s004.tiff]

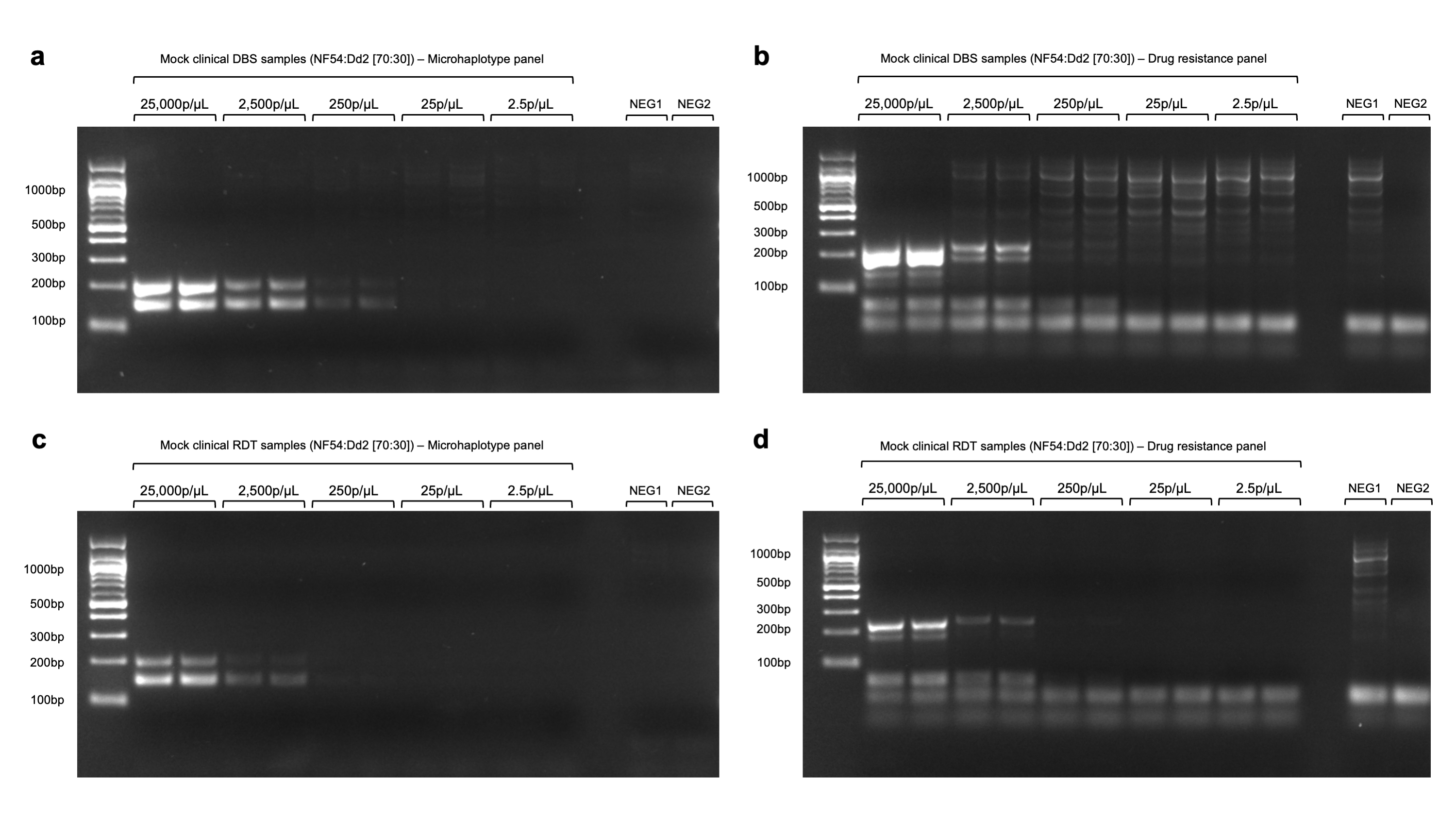

Supplement: S4 Fig — DNA was extracted from 50 μL DBS, or from RDTs with 5 μL blood spotted. a, Microhaplotypes from DBS. b, Drug resistance markers from DBS. c, Microhaplotypes from RDTs. d, Drug resistance markers from RDTs. Mock mixtures were spotted on DBS or RDTs at different parasite densities ranging from 25,000 parasites/μL– 2.5 parasites/μL. For DBS, 50 μL was blotted onto filter papers to mimic DBS samples. For RDTs, 5 μL were blotted onto RDTs to mimic RDT samples. All samples were extracted and assessed in duplicate. For all samples, 3 μL were loaded onto a 2.5% agarose gel and run at 90V for 1 hour. DBS = Dried Blood Spots. RDT = Rapid Diagnostic Test. Neg1 = Negative control (only human whole blood used as template). Neg2 = Negative control (nuclease free water used as template). (TIFF) [file pgph.0002743.s005.tiff]
