# Supplementary material for: Using a mobile nanopore sequencing lab for end-to-end genomic surveillance of Plasmodium falciparum: A feasibility study
Source: PLOS Glob Public Health. 2024 Feb 1;4(2):e0002743. doi: 10.1371/journal.pgph.0002743 (PMC10833559; doi:10.1371/journal.pgph.0002743)
Supplement: S1 Table — Appropriate volume of stock concentration per primer was diluted in NF dH2O (added up to 1mL) to get concentration of final pool. Pool concentrations and final concentrations of each primer are indicated. (DOCX) [file pgph.0002743.s006.docx]

| **Panel** | **Primer** | **Direction** | **Sequence (5' - 3')** | **Source** | **Stock conc. (mM)** | **Conc. in final pool (µM)** | **Final conc. in PCR reaction (µM)** |
| --- | --- | --- | --- | --- | --- | --- | --- |
| Microhaplotype | ama1 | Forward | GAACTCAATATAGACTTCCATCAGG | Holzschuh et al. 2023 | 1 | 5 | 0.1 |
| Microhaplotype | cpmp | Forward | GGAAGCTATAGGTATCAGATCC | Holzschuh et al. 2023 | 1 | 10 | 0.2 |
| Microhaplotype | cpp | Forward | AACACAATCTTCCTTAGCCAATTC | Holzschuh et al. 2023 | 1 | 5 | 0.1 |
| Microhaplotype | csp | Forward | GACCCAAACCGAAATGTAGATG | Holzschuh et al. 2023 | 1 | 5 | 0.1 |
| Microhaplotype | t04 | Forward | CACCAAAATATTATATACCACAAGAC | Holzschuh et al. 2023 (modified from Tessema et al., 2020) | 1 | 15 | 0.3 |
| Microhaplotype | t73 | Forward | CTGGTACTATTATACCATATGTTGC | Holzschuh et al. 2023 (modified from Tessema et al., 2020) | 1 | 10 | 0.2 |
| Microhaplotype | ama1 | Reverse | CCTGCATGTCTTGAACATAAAGTC | Holzschuh et al. 2023 | 1 | 5 | 0.1 |
| Microhaplotype | cpmp | Reverse | TAGAATACGTGCTTTATAAACAAAGAG | Holzschuh et al. 2023 | 1 | 10 | 0.2 |
| Microhaplotype | cpp | Reverse | ATTACTACCTTTCAGCATATCCGA | Holzschuh et al. 2023 | 1 | 5 | 0.1 |
| Microhaplotype | csp | Reverse | GAGCCAGGCTTTATTCTAACTTG | Holzschuh et al. 2023 | 1 | 5 | 0.1 |
| Microhaplotype | t04 | Reverse | GGAAAATCTTTGGTGGGAAAAATAG | Holzschuh et al. 2023 (modified from Tessema et al., 2020) | 1 | 15 | 0.3 |
| Microhaplotype | t73 | Reverse | TCACCAACCTTTTTAGAATCAAGC | Holzschuh et al. 2023 (modified from Tessema et al., 2020) | 1 | 10 | 0.2 |
| Drug resistance | dhfr_t25 | Forward | CTAGGAAATAAAGGAGTATTACCATG | Holzschuh et al. 2023 (modified from Tessema et al., 2020) | 1 | 10 | 0.2 |
| Drug resistance | dhfr_t26 | Forward | TGTTTATATCATTAACAAAGTTGAAGATC | Holzschuh et al. 2023 (modified from Tessema et al., 2020) | 1 | 10 | 0.2 |
| Drug resistance | dhps_436-437 | Forward | GGTGCTAGTGTTATAGATATAGGTGG | LaVerriere et al., 2022 | 1 | 10 | 0.2 |
| Drug resistance | dhps_t49 | Forward | AAAAGAGGAAATCCACATACAATGG | Holzschuh et al. 2023 (modified from Tessema et al., 2020) | 1 | 10 | 0.2 |
| Drug resistance | k13_520-580 | Forward | TCTCTCACCATTAGTTCCACCAA | LaVerriere et al., 2022 | 1 | 15 | 0.3 |
| Drug resistance | mdr1_1034-1042 | Forward | GTAAATGCAGCTTTATGGGGAT | LaVerriere et al., 2022 | 1 | 10 | 0.2 |
| Drug resistance | mdr1_1246 | Forward | CCAATCTGGATCTGCAGAAGA | LaVerriere et al., 2022 | 1 | 5 | 0.1 |
| Drug resistance | mdr1_t34 | Forward | AAATGTTTACCTGCACAACATAGAAA | Holzschuh et al. 2023 (modified from Tessema et al., 2020) | 1 | 10 | 0.2 |
| Drug resistance | mdr1_t35 | Forward | GAACAAGTGAGTTCAGGAATTGG | Holzschuh et al. 2023 (modified from Tessema et al., 2020) | 1 | 5 | 0.1 |
| Drug resistance | mdr2_t96 | Forward | TTTTCTCCACTTTGTAATTTTTATTGTTG | Holzschuh et al. 2023 (modified from Tessema et al., 2020) | 1 | 10 | 0.2 |
| Drug resistance | dhfr_t25 | Reverse | AATATAACATTTATCCTATTGCTTAAAGG | Holzschuh et al. 2023 (modified from Tessema et al., 2020) | 1 | 10 | 0.2 |
| Drug resistance | dhfr_t26 | Reverse | ACATCGCTAACAGAAATAATTTGATAC | Holzschuh et al. 2023 (modified from Tessema et al., 2020) | 1 | 10 | 0.2 |
| Drug resistance | dhps_436-437 | Reverse | ACAGGTACTACTAAATCTCTTTCAC | LaVerriere et al., 2022 | 1 | 10 | 0.2 |
| Drug resistance | dhps_t49 | Reverse | ATTTATTACAACATTTTGATCATTCATGC | Holzschuh et al. 2023 (modified from Tessema et al., 2020) | 1 | 10 | 0.2 |
| Drug resistance | k13_520-580 | Reverse | AAGGCTTTATTTGAAACTGAGGTGT | LaVerriere et al., 2022 | 1 | 15 | 0.3 |
| Drug resistance | mdr1_1034-1042 | Reverse | GAAGGATCCAAACCAATAGGC | LaVerriere et al., 2022 | 1 | 10 | 0.2 |
| Drug resistance | mdr1_1246 | Reverse | ACATGGGTTCTTGACTAACTATTG | LaVerriere et al., 2022 | 1 | 5 | 0.1 |
| Drug resistance | mdr1_t34 | Reverse | GATGTAATTACATCCATACAATAACTTG | Holzschuh et al. 2023 (modified from Tessema et al., 2020) | 1 | 10 | 0.2 |
| Drug resistance | mdr1_t35 | Reverse | TTTCTTATTACATATGACACCACAAAC | Holzschuh et al. 2023 (modified from Tessema et al., 2020) | 1 | 5 | 0.1 |
| Drug resistance | mdr2_t96 | Reverse | GGGTGGTATCATGAGAATAGTTG | Holzschuh et al. 2023 (modified from Tessema et al., 2020) | 1 | 10 | 0.2 |
