# Supplementary material for: Using a mobile nanopore sequencing lab for end-to-end genomic surveillance of Plasmodium falciparum: A feasibility study
Source: PLOS Glob Public Health. 2024 Feb 1;4(2):e0002743. doi: 10.1371/journal.pgph.0002743 (PMC10833559; doi:10.1371/journal.pgph.0002743)
Supplement: S2 Table — (DOCX) [file pgph.0002743.s007.docx]

| **Gene** | **Marker** | **Chromosome** | **Position chromosome** | **Strand** | **Mutation** | **REF** | **ALT** | **Position amplicon** | **Info** |
| --- | --- | --- | --- | --- | --- | --- | --- | --- | --- |
| *Pfdhfr* | DR_t25_dhfr | Pf3D7_04_v3 | 748239 | + | N51I | A | T | 9 |  |
| *Pfdhfr* | DR_t25_dhfr | Pf3D7_04_v3 | 748262 | + | C59R | T | C | 32 |  |
| *Pfdhfr* | DR_t25_dhfr | Pf3D7_04_v3 | 748410 | + | S108N | G | A | 180 |  |
| *Pfdhfr* | DR_t25_dhfr | Pf3D7_04_v3 | 748410 | + | S108T | G | C | 180 |  |
| *Pfdhfr* | DR_t26_dhfr | Pf3D7_04_v3 | 748577 | + | I164L | A | T | 45 |  |
| *Pfmdr1* | DR_t34_mdr1 | Pf3D7_05_v3 | 958145 | + | N86Y | A | T | 92 | 86Y encoded by **T**AT |
| *Pfmdr1* | DR_t34_mdr1 | Pf3D7_05_v3 | 958146 | + | N86F | A | T | 93 | 86F encoded by **TT**T |
| *Pfmdr1* | DR_t35_mdr1 | Pf3D7_05_v3 | 958440 | + | Y184F | A | T | 51 |  |
| *Pfmdr1* | DR_mdr1_1034-1042 | Pf3D7_05_v3 | 960989 | + | S1034C | A | T | 3 |  |
| *Pfmdr1* | DR_mdr1_1034-1042 | Pf3D7_05_v3 | 961013 | + | N1042D | A | G | 27 |  |
| *Pfmdr1* | DR_mdr1_1246 | Pf3D7_05_v3 | 961625 | + | D1246Y | G | T | 71 |  |
| *Pfdhps* | DR_dhps_436-437 | Pf3D7_08_v3 | 549681 | + | S436A | T | G | 8 |  |
| *Pfdhps* | DR_dhps_436-437 | Pf3D7_08_v3 | 549682 | + | S436A | C | T | 9 |  |
| *Pfdhps* | DR_dhps_436-437 | Pf3D7_08_v3 | 549685 | + | G437A | G | C | 12 | **G**437A the ref is resistant (rewritten as A437**G)** |
| *Pfdhps* | DR_t49_dhps | Pf3D7_08_v3 | 549993 | + | K540E | A | G | 3 |  |
| *Pfdhps* | DR_t49_dhps | Pf3D7_08_v3 | 550117 | + | A581G | C | G | 127 |  |
| *Pfdhps* | DR_t49_dhps | Pf3D7_08_v3 | 550212 | + | A613T | G | A | 222 |  |
| *Pfdhps* | DR_t49_dhps | Pf3D7_08_v3 | 550212 | + | A613S | G | T | 222 |  |
| *Pfk13* | DR_k13_520-580 | Pf3D7_13_v3 | 1725247 | - | D584V | A | T | 220 |  |
| *Pfk13* | DR_k13_520-580 | Pf3D7_13_v3 | 1725259 | - | C580Y | G | A | 208 |  |
| *Pfk13* | DR_k13_520-580 | Pf3D7_13_v3 | 1725266 | - | A578S | G | T | 201 |  |
| *Pfk13* | DR_k13_520-580 | Pf3D7_13_v3 | 1725277 | - | P574L | C | T | 190 |  |
| *Pfk13* | DR_k13_520-580 | Pf3D7_13_v3 | 1725295 | - | V568G | T | G | 172 |  |
| *Pfk13* | DR_k13_520-580 | Pf3D7_13_v3 | 1725316 | - | R561H | G | A | 151 |  |
| *Pfk13* | DR_k13_520-580 | Pf3D7_13_v3 | 1725340 | - | P553L | C | T | 127 |  |
| *Pfk13* | DR_k13_520-580 | Pf3D7_13_v3 | 1725370 | - | I543T | A | G | 97 |  |
| *Pfk13* | DR_k13_520-580 | Pf3D7_13_v3 | 1725382 | - | R539T | C | G | 85 |  |
| *Pfk13* | DR_k13_520-580 | Pf3D7_13_v3 | 1725385 | - | G538V | G | T | 82 |  |
| *Pfk13* | DR_k13_520-580 | Pf3D7_13_v3 | 1725388 | - | N537I | A | T | 79 |  |
| *Pfk13* | DR_k13_520-580 | Pf3D7_13_v3 | 1725418 | - | P527H | C | A | 49 |  |
| *Pfk13* | DR_k13_520-580 | Pf3D7_13_v3 | 1725454 | - | R515K | G | A | 13 |  |
| *Pfmdr2* | DR_t96_mdr2 | Pf3D7_14_v3 | 1956202 | - | I492V | A | G | 87 |  |
| *Pfmdr2* | DR_t96_mdr2 | Pf3D7_14_v3 | 1956225 | - | T484I | C | T | 64 |  |
