# Supplementary material for: Using a mobile nanopore sequencing lab for end-to-end genomic surveillance of Plasmodium falciparum: A feasibility study
Source: PLOS Glob Public Health. 2024 Feb 1;4(2):e0002743. doi: 10.1371/journal.pgph.0002743 (PMC10833559; doi:10.1371/journal.pgph.0002743)
Supplement: S3 Table — (DOCX) [file pgph.0002743.s008.docx]

| **Step** | **Temp** | **Time** | **Cycles** |
| --- | --- | --- | --- |
| Initial Denaturation | 95°C | 10 min | 1 |
| Denaturation | 98°C | 15 sec | 35 |
| Annealing | 58°C | 15 sec |  |
| Extension | 72°C | 30 sec |  |
| Final Extension | 72°C | 2 min | 1 |
| Hold | 4°C | ∞ |  |
