# Supplementary material for: Using a mobile nanopore sequencing lab for end-to-end genomic surveillance of Plasmodium falciparum: A feasibility study
Source: PLOS Glob Public Health. 2024 Feb 1;4(2):e0002743. doi: 10.1371/journal.pgph.0002743 (PMC10833559; doi:10.1371/journal.pgph.0002743)
Supplement: S4 Table — Magharibi ‘B’ and Mjini are highlighted in orange and purple, respectively. Source: ZAMEP. (DOCX) [file pgph.0002743.s009.docx]

| **Year** | **2013** | **2014** | **2015** | **2016** | **2017** | **2018** | **2019** | **2020** | **2021** |
| --- | --- | --- | --- | --- | --- | --- | --- | --- | --- |
| **Kaskazini ‘A’** | 280 | 474 | 608 | 270 | 364 | 525 | 600 | 974 | 430 |
| **Kaskazini ‘B’** | 252 | 504 | 477 | 355 | 348 | 514 | 466 | 730 | 375 |
| **Kati** | 512 | 629 | 723 | 454 | 763 | 761 | 944 | 1553 | 997 |
| **Kusini** | 325 | 370 | 327 | 151 | 312 | 267 | 238 | 484 | 511 |
| **Magharibi ‘A’** | 165 | 322 | 456 | 380 | 427 | 704 | 954 | 1276 | 823 |
| **Magharibi ‘B’** | 588 | 721 | 949 | 536 | 790 | 932 | 1462 | 2709 | 1605 |
| **Mjini** | 265 | 442 | 766 | 580 | 745 | 1189 | 2038 | 4551 | 1262 |
| **Unguja** | 2387 | 3462 | 4306 | 2726 | 3749 | 4892 | 6702 | 12277 | 6003 |
| **Micheweni** | 258 | 290 | 413 | 418 | 398 | 554 | 339 | 650 | 135 |
| **Mkoani** | 140 | 155 | 156 | 128 | 98 | 151 | 207 | 249 | 94 |
| **Wete** | 269 | 207 | 189 | 274 | 176 | 304 | 340 | 648 | 247 |
| **Chakechake** | 54 | 161 | 135 | 103 | 134 | 189 | 185 | 317 | 208 |
| **Pemba** | 721 | 813 | 893 | 923 | 806 | 1198 | 1071 | 1864 | 684 |
| **Grand Total** | 3108 | 4275 | 5199 | 3649 | 4555 | 6090 | 7773 | 14141 | 6687 |
