# Supplementary material for: Using a mobile nanopore sequencing lab for end-to-end genomic surveillance of Plasmodium falciparum: A feasibility study
Source: PLOS Glob Public Health. 2024 Feb 1;4(2):e0002743. doi: 10.1371/journal.pgph.0002743 (PMC10833559; doi:10.1371/journal.pgph.0002743)
Supplement: S5 Table — (DOCX) [file pgph.0002743.s010.docx]

| **Item** | **Vendor/Cat #** | **Quantity** | **Storage** | **Purpose** | **Comments** |
| --- | --- | --- | --- | --- | --- |
| Whatman 3mm paper | VWR/3030-153 | 1 |  | Sample collection |  |
| Silica Gel Desiccant | VWR/136280-250G | 1 |  | Sample collection |  |
| Zipper plastic bags | KCH/5101 | 3 |  | Sample collection |  |
| Microtainer Contact-activated lancet | VWR/89027-103 | 1 |  | Sample collection |  |
| MinION Mk1C | VWR/76487-170 | 1 |  | Nanopore sequencing |  |
| MinION R10.4 flow cell | VWR/76519-502 | 2 | 4-8°C | Nanopore sequencing |  |
| Native Barcoding Kit 24 | VWR/76519-506 | 1 | -20°C | Nanopore sequencing |  |
| Kit Flow Cell Wash 6 Reactions | VWR/76487-116 | 1 | -20°C | Nanopore sequencing |  |
| Sequencing Auxiliary Vials | VWR/76519-514 | 1 | -20°C | Nanopore sequencing |  |
| Blunt/TA Ligase Master Mix | New England Biolabs/M0367S | 3 | -20°C | Nanopore sequencing |  |
| Blunt/TA Ligase Master Mix | New England Biolabs/M0367L | 1 | -20°C | Nanopore sequencing |  |
| NEBNext® Ultra™ II End Repair/dA-Tailing Module | New England Biolabs/E7546S | 1 | -20°C | Nanopore sequencing |  |
| NEBNext® Quick Ligation Module | New England Biolabs/E6056S | 1 | -20°C | Nanopore sequencing |  |
| CleanNGS | Bulldog Bio/CNGS050 | 1 | 4-8°C | Nanopore sequencing |  |
| Qubit™ 4 | ThermoScientific/Q33226 | 1 |  | Nanopore sequencing |  |
| 1X dsDNA BR Working Solution | ThermoScientific/Q33262 | 1 |  | Nanopore sequencing |  |
| Qubit™ 1X dsDNA BR Assay Standards | ThermoScientific/Q33261 | 1 | 4-8°C | Nanopore sequencing |  |
| BOMB microtube rack (3D printed) | N.A./N.A. | 3 |  | Nanopore Sequencing | <https://bomb.bio/protocols/> |
| LoBind Microcentrifuge Tubes 1.5mL (50 tubes each) | Eppendorf/022431021 | 10 bags |  | Nanopore sequencing |  |
| 2TB Extreme Portable SSD | SanDisk | 1 |  | Nanopore sequencing |  |
| PCR tubes 0.5mL | VWR/10011-830 | 1 bag |  | Multiplex PCR/Nanopore sequencing |  |
| 0.2mL PCR strip with caps | VWR/20170-004 | 2 |  | Multiplex PCR/Nanopore sequencing |  |
| Bento Lab | bento/N.A. | 1 |  | Multiplex PCR/DNA extraction |  |
| KAPA HiFi HotStart PCR Kit | Roche/KK2502 | 2 | -20°C | Multiplex PCR |  |
| PCR grade water 2mL | bento/PGW-2 | 13 |  | Multiplex PCR |  |
| Agarose tablets (200 tablets) | bento/N.A. | 1 box |  | Multiplex PCR |  |
| GelGreen DNA stain 500uL | bento/GG-500 | 1 |  | Multiplex PCR |  |
| TBE electrophoresis buffer 1000mL | bento/TBE-1000 | 1 |  | Multiplex PCR |  |
| 100 bp DNA ladder | bento/DL-100BP-500 | 1 | -20°C | Multiplex PCR |  |
| Gel loading dye, blue (6X) 1mL | bento/LD-BL-1 | 5 |  | Multiplex PCR |  |
| Primer stock tubes | Sigma-Aldrich/VC00021 | 1 each | -20°C | Multiplex PCR |  |
| Primer working solution in water | N.A./N.A. | 5 tubes | -20°C | Multiplex PCR |  |
| P. fal DNA (positive control) | From lab stock/N.A. | 1 tube | -20°C | Multiplex PCR |  |
| PCR Cooler, 96-well | VWR/76343-420 | 1 |  | Multiplex PCR |  |
| Mini Block Heater | VWR/10153-320 | 1 |  | DNA extraction/Nanpore sequencing |  |
| Phosphate Buffered Saline (PBS) | VWR/97063-658 | 1 |  | DNA extraction |  |
| Water, sterile, nuclease-free | VWR/97062-794 | 1 |  | DNA extraction |  |
| Chelex® 100 sodium form | Sigma-Aldrich/C7901-25G | 1 |  | DNA extraction |  |
| TWEEN® 20 | Sigma-Aldrich/P1379-25ML | 1 |  | DNA extraction |  |
| 0.8mL 96-well storage plates | ThermoScientific/AB0765 | 2 |  | DNA extraction |  |
| Storage plate 8 cap strips | ThermoScientific/AB0981 | 2 |  | DNA extraction |  |
| Scissors | VWR/82027-588 | 2 |  | DNA extraction |  |
| Foreceps | VWR/82027-438 | 2 |  | DNA extraction |  |
| 1.5mL Eppendorf tubes | USA Scientific/1615-5500 | 1 bag |  | DNA extraction |  |
| 50mL falcon tubes | VWR/10026-078 | 10 tubes |  | DNA extraction |  |
| Kimtech Kimwipes | VWR/89218-057 | 2 |  | DNA extraction |  |
| Parafilm | VWR/52858-000 | 1 |  | DNA extraction |  |
| Glass bottle 250mL | VWR/10754-816 | 2 |  | DNA extraction |  |
| Mini Vortexer | VWR/470230-486 | 1 |  | All steps |  |
| Mini Microcentrifuge | VWR/76163-552 | 1 |  | All steps |  |
| Pipette (100 - 1000uL) | USA Scientific/7110-1000 | 1 |  | All steps |  |
| Pipette (20 - 200uL) | bento/A3026 | 2 |  | All steps |  |
| Pipette (2 - 20uL) | USA Scientific/7100-0221 | 1 |  | All steps |  |
| Pipet tips filter - 10uL | USA Scientific/1120-3710 | 2 boxes |  | All steps |  |
| Pipet tips filter - 300uL | USA Scientific/1120-9710 | 2 boxes |  | All steps |  |
| Pipet tips filter - 1000uL | USA Scientific/1122-1730 | 2 boxes |  | All steps |  |
| Pipet tips non-filter - 10uL | USA Scientific/1110-3000 | 2 bags |  | All steps |  |
| Pipet tips non-filter - 300uL | USA Scientific/1110-9000 | 1 bag |  | All steps |  |
| Pipet tips non-filter - 1000uL | USA Scientific/1112-1020 | 1 bag |  | All steps |  |
| Lab coat | VWR/10753-936 | 1 |  | All steps |  |
| Nitrile gloves Large | USA Scientific/3915-4400 | 2 |  | All steps |  |
