# Supplementary material for: Using a mobile nanopore sequencing lab for end-to-end genomic surveillance of Plasmodium falciparum: A feasibility study
Source: PLOS Glob Public Health. 2024 Feb 1;4(2):e0002743. doi: 10.1371/journal.pgph.0002743 (PMC10833559; doi:10.1371/journal.pgph.0002743)
Supplement: S6 Table — (DOCX) [file pgph.0002743.s011.docx]

| Gene name (*PlasmoDB* ID) | Key mutations targeted for genotyping | Number of amplicons | Associated antimalarial resistance |
| --- | --- | --- | --- |
| Dihydrofolate reductase, *dhfr*  (PF3D7_0417200) | N51I, C59R, S108N, I164L | 2 | Pyrimethamine resistance markers |
| Dihydropteroate synthase, *dhps*  (PF3D7_0810800) | S436A, A437G, K540E, A581G, A613S/T | 2 | Sulfadoxine resistance markers |
| Multidrug resistance protein 1, *mdr1*  (PF3D7_0523000) | N86Y, N86F, Y184F, S1034C, N1042D, D1246Y | 4 | No direct inferences, but associated with resistance to several antimalarials including lumefantrine and amodiaquine |
| Multidrug resistance protein 2, *mdr2*  (PF3D7_1447900) | T484I, I492V | 1 | No direct inferences, but suggested to be involved in artemisinin partial resistance |
| kelch13, *k13*  (PF3D7_1343700) | Different mutations in the propeller domain: R515K, S522C, P527L, N537I, N537D, G538V, R539T, I543T, P553L, R561H, V568G, P574L, R575K, M579I, C580Y, D584V | 1 | Artemisinin partial resistance markers |
