# Supplementary material for: Using a mobile nanopore sequencing lab for end-to-end genomic surveillance of Plasmodium falciparum: A feasibility study
Source: PLOS Glob Public Health. 2024 Feb 1;4(2):e0002743. doi: 10.1371/journal.pgph.0002743 (PMC10833559; doi:10.1371/journal.pgph.0002743)
Supplement: S7 Table — (DOCX) [file pgph.0002743.s012.docx]

| Gene name (*PlasmoDB* ID) | Number of amplicons | Associated phenotype |
| --- | --- | --- |
| Apical membrane antigen 1, *ama1* (PF3D7_1133400) | 1 | Vaccine candidate antigen; potential for use as a marker of complexity of infection |
| Conserved Plasmodium membrane protein, *cpmp* (PF3D7_0104100) | 1 | Potential for use as a marker of complexity of infection |
| Conserved Plasmodium protein, *cpp* (PF3D7_1475800) | 1 | Potential for use as a marker of complexity of infection |
| Circumsporozoite protein, *csp* (PF3D7_0304600) | 1 | Leading vaccine and monoclonal antibody target antigen; potential for use as a marker of complexity of infection |
| Surface-associated interspersed protein 1.1, *surf1.1* (PF3D7_0113100) | 1 | Potential for use as a marker of complexity of infection |
| Cell traversal protein for ookinetes and sporozoites, *celtos*  (PF3D7_1216600) | 1 | Vaccine candidate antigen, potential for use as a marker of complexity of infection |
