# Supplementary material for: Using a mobile nanopore sequencing lab for end-to-end genomic surveillance of Plasmodium falciparum: A feasibility study
Source: PLOS Glob Public Health. 2024 Feb 1;4(2):e0002743. doi: 10.1371/journal.pgph.0002743 (PMC10833559; doi:10.1371/journal.pgph.0002743)
Supplement: S8 Table — (DOCX) [file pgph.0002743.s013.docx]

| Run | Flow cell | Samples per run (of which field samples) | Run time | Output (Gb) | Total reads | Mean  Q-score | Median Q-score | Reads with ≥Q15, pass (%) | DNA extraction method |
| --- | --- | --- | --- | --- | --- | --- | --- | --- | --- |
| R9.4.1 – Run1 | R9.4.1 | 4 (0) | 5h 07m | 7.5 | 490,754 | 12.9 | 13.3 | 136219 (27.8%) | NucleoMag |
| R9.4.1 – Run2 | R9.4.1 | 9 (0) | 4h 02m | 12.0 | 658,399 | 13.2 | 13.5 | 216782 (32.9%) | NucleoMag |
| R9.4.1 – Run3 | R9.4.1 | 6 (0) | 4h 16m | 3.7 | 338,968 | 12.3 | 12.7 | 72859 (21.5%) | NucleoMag |
| R10.4 – Run1 | R10.4 | 10 (0) | 2h 37m | 11.0 | 828,118 | 14.4 | 15.1 | 423932 (51.2%) | NucleoMag |
| R10.4 – Run2 | R10.4 | 10 (0) | 6h 32m | 8.8 | 632,594 | 14.2 | 15.0 | 313071 (49.5%) | NucleoMag |
| R10.4 – Run3* | R10.4 | 24 (10) | 5h 04m | 14.9 | 1,150,548 | 12.2 | 12.9 | 332715 (28.9%) | (NucleoMag), Tween-Chelex |
| R10.4 – Run4** | R10.4 | 12 (0) | 25h 20min | 8.5 | 676,193 | 11.0 | 10.9 | 150573 (22.3%) | NucleoMag |
| R10.4 – Run5** | R10.4 | 13 (0) | 21h 32min | 9.6 | 724,498 | 11.9 | 12.5 | 195662 (27.0%) | NucleoMag |
| R10.4 – Run6 | R10.4 | 20 (0) | 6h 26min | 8.1 | 581,023 | 12.1 | 12.8 | 176179 (30.3%) | Tween-Chelex |
| R10.4 – Run7** | R10.4 | 20 (0) | 31h 30min | 14.6 | 912,122 | 12.7 | 13.4 | 336210 (36.9%) | Tween-Chelex |
| R10.4 – Field1 | R10.4 | 24 (20) | 4h 18min | 18.3 | 1,357,844 | 12.6 | 13.2 | 487619 (35.9%) | Tween-Chelex |
| R10.4 – Field2 | R10.4 | 24 (22) | 6h 09min | 15.2 | 1,059,636 | 13.3 | 14.2 | 446685 (42.2%) | Tween-Chelex |

*10 field DBS samples from previous study (Holzschuh et al., 2023 Nat Comms) were already extracted using NucleoMag; all other control samples extracted using Tween-Chelex.

**Flow cells with <350 pores left.
