# Supplementary material for: Using a mobile nanopore sequencing lab for end-to-end genomic surveillance of Plasmodium falciparum: A feasibility study
Source: PLOS Glob Public Health. 2024 Feb 1;4(2):e0002743. doi: 10.1371/journal.pgph.0002743 (PMC10833559; doi:10.1371/journal.pgph.0002743)
Supplement: Attachment — Submitted filename: Response_to_Reviewers.docx [file pgph.0002743.s014.docx]

**Point-by-point responses to reviewer comments**

Holzschuh et al., “Real-time genomic surveillance of Plasmodium falciparum using a mobile nanopore sequencing lab: a feasibility study”

We thank the editors and reviewers for their helpful comments and suggestions for improvement. Original reviewer comments are numbered and in italics, and our responses are indented and in normal font. Additionally, changes to the text are indicated throughout this document and the manuscript in red text. We believe that the changes made in response to these suggestions have led to a substantially improved manuscript.

**Reviewer #1:**

*The authors developed and validated a two-multiplexed amplicon P. falciparum sequencing assay using the MinION that incorporated 6 microhaplotypes and ten drug resistance markers. This assay was then tested in a mobile lab setting in Zanzibar.*

*Overall, this is a valuable pilot study bringing a proof-of-concept of using the MinION (and a mobile lab) to generate valuable data in country also highlighting potential challenges of this approach.*

*1) Data availability – currently pending so not (yet) accessible?*

**RESPONSE:** At the time of the submission, the repository had not yet been finalized. All sequence data and code used in this analysis can be found at https://doi.org/10.5281/zenodo.10086238.

*2) Title: this is set as real-time genomic surveillance and although the technology does show fast results, I would argue these not to be real-time (the paper states “actionable data within a few days”). The authors could amend their title or maybe clarify the real-time aspect of it further.*

**RESPONSE:**

We have opted to change the title to “Using a mobile nanopore sequencing lab for end-to-end genomic surveillance of *Plasmodium falciparum*: a feasibility study”

*3) The authors state they developed a pipeline that allows genotyping of polyclonal infections. Technically I did not see a pipeline (maybe one could call it a framework?). I was not able to access the code (related to point 1) – but depending on how the code is shared and commented on, the author could refer to the code repository in the method section of this paper. This would remove the need to add code snippets and keep the method section clean (i.e. just note which software was used and then refer to code).*

**RESPONSE:**

We have changed the wording from “pipeline” to “[analysis] workflow” and have changed this throughout the manuscript.

All basecalled sequence data and code used in this analysis can be found at https://doi.org/10.5281/ zenodo.10086238.

We chose to keep the code snippets in the methods; however, we have simplified them in the manuscript as much as possible.

*4) Could the authors clarify as to why they have limited the sequencing to 16 markers and why they chose the markers they did? Is there an option to extend? If yes - that would be valuable to highlight. Was it two multiplexed to evaluate the barcode cross-talk? Is there the possibility to put it into one multiplex?*

**RESPONSE:**

Our initial protocol with 35 markers follows a novel approach for amplification in microdroplets (ddPCR AmpSeq; see *Holzschuh et al., 2023 Nat Commun*). This approach requires specific equipment not typically available in a field laboratory (i.e., a droplet generator). For a simplified protocol, we selected 6 diverse markers (with high H_E_) that can be used to determine MOI, genetic diversity, and whether infections in a cluster are related.

We developed a separate panel for the drug resistance markers to allow a modular approach, e.g., if only drug resistance data is required, no changes to the protocol need to be made.

While running two pools gave us the opportunity to assess barcode crosstalk, this was an added benefit but not the motivation to develop two pools. This was a proof-of-concept study with the primary goal of evaluating AmpSeq using a mobile lab in an endemic setting rather than multiplexing as many markers as possible. We could probably include more markers in a single reaction, but with many more markers, the main limitation is removing all primer dimer (which is difficult with such short amplicons).

However, with the knowledge gained from this study, we are now working to develop more nanopore assays for different use cases.

We have added the following to the methods section to clarify (page 8, lines 184-187):

“We developed two multiplex PCR panels, a 6-plex of highly polymorphic microhaplotypes to estimate multiplicity of infection (MOI) and genetic diversity, previously used in Zanzibar (6), and a 10-plex of known antimalarial drug resistance markers. The two panels can be used independently from each other, depending on the use case.”

*5) Differences in chemistry – as ONT states that their new chemistry comes with better quality (and more data), it might be worth clarifying the differences in the data analysis (i.e. separating outcomes in regards to median quality, run time, read amount etc between old and new chemistry). Since the old chemistry will no longer be supported or sold by ONT, it would be interesting to showcase the new chemistry results more (are they actually much better?).*

**RESPONSE:**

Table S8 shows that unfortunately there was not much improvement from R9.4.1 to R10.4 flow cells. We only have very little data from the R9.4.1 flow cell, which prevents a meaningful comparison between the two. We therefore think it would not add anything to the manuscript to add more than is already added to the current manuscript.

In addition, it should be noted that the Kit 12 and R10.4 flow cell used in this study are already obsolete. The latest Kit 14 and R10.4.1 flow cells are said to improve accuracy. In addition, we used the Guppy basecaller, and now the Dorado is available, which is also supposed to improve basecalling accuracy.

We have added a sentence on dorado in the discussion (page 25, lines 622-624):

“In addition, ONT’s new basecalling software *Dorado* (<https://github.com/nanoporetech/dorado/>) is expected to further improve raw read accuracy and overall performance.”

*6) the authors have calculated a cut-off of >65 reads per haplotype. If I understood it correctly, they used all samples in one pot to calculate this. I wonder if it would have been more suitable to create a measurement that was relative to the depth of the sample. In our experience the sequencing tends to be uneven between the samples so a hard cut-off could lead to a loss of valuable data in those samples with a lower read amount.*

**RESPONSE:** The purpose of the read per haplotype cut-off of >65 was to remove reads originating from cross-talk events. In addition to the 65 reads cut-off, we also apply a within-host haplotype frequency cut-off of ≥2% that is relative to the read depth,

In our data, the cross-talk events seemed to be consistent across the different sequencing runs, with an interquartile range (IQR) of 20-62 reads per cross-talk event. Other users of our protocol might lower (or increase) the reads per haplotype cut-off if they observe a different number of sequence reads originating from cross-talk events. This might also improve/change in the future with the release of new kits and flow cells. We agree that we might miss some haplotypes in samples (especially low-density samples) with a lower number of reads, but we decided to apply this rather stringent cut-off to protect against false-positive haplotypes at the expense of some sensitivity. Figure 4 nicely shows increased sensitivity when no cut-off is applied (but at the expense of specificity). With control samples, both panels showed good sensitivity in recovering minority clone haplotypes (Table 2 and Fig.4 for drug resistance marker, Table 3 and Fig. 4 for microhaplotypes).

We are also optimistic that future improvements in kits and flow cells, as well as more accurate base callers, will allow users to lower this threshold and increase sensitivity without sacrificing specificity.

*7) Figure 1: both Unguja and Pemba are in a square – maybe add the names to the picture or clarify the border in the legend.*

**RESPONSE:** We have slightly changed the border in Figure 1 to show only Unguja (not the whole Zanzibar archipelago).

*8) Figure S3: the y-axis labelling is slightly confusing. The axis is showing the nr of reads not the log10 nr of reads. I think it is clearly visible that it was plotted using a log10 scale so could possibly just remove from label?*

**RESPONSE:** We have changed the y-axis label to “Number of reads” (it is still mentioned in the figure legend that it is plotted using a log10 scale)

*9) Figure 5 – see point 8– misleading axis label*

**RESPONSE:** Again, y-axis label was changed to “Number of reads”.

*10) general note: the authors are losing a lot of data during their Q15 filter step (over 50%), it might be worth exploring a slightly lower threshold. Nanopore recommends 7, though most I know filter at around 12.*

**RESPONSE:**

We agree that we lose quite many reads, however, most importantly, the DADA2 software requires high quality reads (see here: <https://github.com/benjjneb/dada2/issues/759>). In the past, DADA2 could not be used to analyze amplicon sequencing data originating from nanopore sequencing due to their low quality. We therefore decided to use a higher Q threshold as the main aim was to decrease noise in the data.

Further, due to many low complex sequences in the *P. falciparum* genome (highly A/T-rich, homopolymeric regions, etc), we decided to use a higher threshold of Q15 to remove erroneous reads, as nanopore sequencing still struggles with homopolymeric regions, for example. If we lowered the Q threshold, most of these reads would probably be removed during haplotype calling. Also, Q15 is still rather low in terms of base call accuracy compared to Illumina sequencing (Q15 only equals a base call accuracy of ~96.8%; compared to >99.9% for Illumina).

We have added the following to the methods (page 10, lines 243-247the success):

“Due to the high-quality reads required by the DADA2 software, the unusual genome structure of P. falciparum, particularly the high A/T content (81%), and the expanses of repetitive, low-complexity regions, that are widely distributed throughout the genome (50), the ~~M~~minimum Q-score for passing reads was increased to a more stringent value of 15 to minimize erroneous~~r rate in the~~ reads.”

**Reviewer #2:**

*Holzschuh et al provide an excellent study addressing the need for field-deployable sequencing assays for ongoing malaria surveillance with the goal to achieve malaria elimination. Without appropriate tools to track malaria transmission and drug resistance, the battle to eliminate malaria will continue. Holzschuh et al choose nanopore technology to address the lack of sequencing throughout Zanzibar. Nanopore is an appropriate sequencing method for this purpose by providing intact long reads, faster sequencing turn-around time but also a cost-effective alternative to short read Illumina sequencing platforms. The small, portable nature of Nanopore, MinION makes it an appealing technology to use for parasite surveillance. This was a proof-of-concept, pilot study to determine whether it would be possible to identify known drug resistant haplotypes quickly and easily. Table 5 clearly identifies the challenges associated around in-country sequencing to which many have struggled with previously including similar studies that have been conducted in Papua New Guinea (PNG). The authors have correctly identified key problems and attempted to identify solutions that could be implemented which seem feasible. Overall, capacity building/strengthening is imperative to the success of genomic surveillance in-country. Similar concepts are being established in other malaria endemic countries. This is a great initiative with huge potential and vital to the success of malaria elimination as drug resistance is increasing.*

*11) Consistent with other studies, Holzschuh et al also had difficulty with roughly half of DBS and RDTs collected in the Zanzibar pilot study had low parasite density and therefore were not sequenced. Would be great for teams across the world to come together and determine how to sequence these low parasite density samples as it would be interesting to see what’s happening at these low levels. Something that could strengthen the paper is to discuss ideas on how to increase the number of samples of low parasite density getting to sequencing?*

**RESPONSE:** We agree that low-density samples are a major challenge to sequence. We have added to the discussion about ideas that could increase the number of samples of low parasite density getting to sequencing (pages 26-27, lines 651-657).

“The success of sequencing low-density parasite samples depends on the amount of template DNA available. Ways to potentially increase the number of samples successfully sequenced include, but are not limited to, DNA enrichment by selective whole genome amplification, increasing the volume of blood collected (i.e., collect as whole blood stored in EDTA tubes), using a more sensitive DNA extraction method, concentrating the DNA (e.g., elute in smaller volume or use SpeedVac), or increasing PCR cycles.”

Some of those methods, however, are not feasible in large-scale studies (e.g., only collection of DBS might be feasible), or in low-resource lab settings (e.g. no SpeedVac available).

AmpSeq in itself is also a way of enriching specific target regions before sequencing.

*12) Discussion was had about the issues around basecalling and haplotype calling. These informatic steps require significant computing capacity. Unsurprisingly authors were faced with poor internet for transferring the data. Should the authors wish to continue malaria surveillance in Zanzibar I believe their statement around a powerful GPU computer would be an appropriate investment. A laptop would have some computing restrictions and not be the most appropriate, although convenient would not save time and result it storage limitations or restricted GPU capacity. However, setting up a high range desktop computer in the ZAMEP laboratory and using hard drives to transfer data would be the best option. For example, you could get an Intel Core-i9 13900KS processor, RTX4090 graphics, 128GB DDR5 RAM and over 25TB of storage for around $5000-$8000. This would be an exceptional investment for the lab. You could collect your samples from various sites around Zanzibar, use the mobile lab for processing samples, sequence with MinION and put data output straight onto the computer to analyse. This would save time and most likely not rely on internet connection or the need for a HPC system. The caveat here would be that someone needs to understand the bioinformatics component. This section of the discussion could be improved with the above information or equivalent.*

**RESPONSE:** Thank you for the great input! We agree and have added to the discussion (page 26, lines 637-644):

“~~Alternatively, a powerful GPU computer, such as a commercial gaming laptop, could be used, adding additional costs and most likely increasing computing time.~~ Investing in a powerful computer would be an appropriate option, allowing the use of the most accurate basecalling models. While a commercial gaming laptop may offer some convenience and would be comparatively inexpensive, it has storage and GPU limitations. Setting up a high-performance desktop computer with GPUs, costing around US$3,000 to US$8,000, and using hard drives to transfer raw sequencing data would be the best option. This investment would enhance the mobile nanopore sequencing lab, saving time and reducing dependence on internet connectivity and HPC clusters.”

*13) Great to see the team thinking ahead. There a several groups currently working on a graphical user interface tool to make analysis quicker and easier for these in-field studies and surveillance of malaria. Regardless of what app is developed, the bottleneck will always be raw sequence data processing but will be beneficial for quick analysis and visual interpretation of a parasite population or drug resistant mutations.*

**RESPONSE:** We very much agree with the reviewer. We have added a little bit on this to the discussion (page 25, lines 607-609):

“~~In the future, t~~The future goal should be to implement the workflow ~~pipeline~~ into an easy-to-use graphical user interface tool to allow rapid analysis and visual interpretation of parasite populations or drug resistance mutations.”

*14) The authors have highlighted the costs of using Nanopore technology. Providing there were going to be a lot of parasite surveillance, I believe you could bulk order flow cells and reduce the cost to $600 per flow cell. It would also be really good to have explored the idea of reusing flow cells. Washing the flow cell does not guarantee the previous template has been removed. Has there been a feasibility study conducted on whether previous sequence samples are contaminating the new sequencing run? Would this then change the results and give false positives ect?*

**RESPONSE:**

We agree, bulk ordering flow cells would reduce the cost quite a bit (from $900 per flow cell to $475 each when ordering 96 flow cells). We have added this to the discussion (page 28, lines 694-695):

“However, by ordering flow cells in bulk, costs can be significantly reduced.”

Regarding reuse of flow cells: ONT claims that the wash kit (with DNase I) should remove 99.9% of the library, so ~0.1% could be contamination (source: ONT documentation Flow Cell Wash Kit (EXP-WSH004)). Furthermore, Lipworth et al. found only 0.02% (147/ 818,091 reads) cross-contamination between subsequent runs on the same flow cell (https://www.ncbi.nlm.nih.gov/pmc/articles/PMC7725331/). With our cutoffs applied and at such a low frequency, it is likely that these potentially contaminating reads are really just adding a little more noise to the data.

Ideally, however, the best solution would be to use the 96 barcode kit instead of the 24 barcode kit. This way, one could use different barcodes in all subsequent runs, or simply multiplex up to 96 different samples in one run (which would prevent reuse of the flow cell anyway). An actual experiment designed to look at this would be best but is beyond the scope of the paper.

We have added this to the discussion (page 28, lines 690-692):

“ONTs washing procedure for reusing flow cells has been shown to be very effective with less than 0.1% of any previously loaded sample contaminating a subsequent run (62).”

Minor changes:

15) Line 225: would be good in the discussion to add a couple of points on re-using flow cells. Typically for most accurate results, flow cells aren’t reused. Would be good to make a statement or direct to publication that has tested the success of re-used flow cells compared to unused.

**RESPONSE:** See response to comment 14) above. We have added the following publication that assessed cross-contamination between subsequent runs (<https://www.ncbi.nlm.nih.gov/pmc/articles/PMC7725331/>).

16) In the methods: it would improve the clarity of the paper to include a couple of really clear statements identifying where data analysis was conducted. It is my understanding that the validation work was conducted at the University of Notre Dame and the pilot study conducted in ZAMEP lab. In the discussion it is discussed issue around transferring data to HPCs. In the methods it should be clearly stated that the mk1c was not used for basecalling but instead a HPC was used and where this is.

**RESPONSE:** The reviewer is correct. All validation work was conducted at the University of Notre Dame. The pilot study was conducted at the laboratory of ZAMEP and included the entire wet lab work. Data was then uploaded (at ZAMEP) to the HPCs of the Center for Research Computing (CRC), University of Notre Dame. We have added to the methods that basecalling was not done on the Mk1C since we wanted to use the highest accuracy basecalling algorithm (‘sup’).

We have added the following to clarify this (page 10, lines 248-252):

“Basecalling was not performed directly on the MinION Mk1C device but instead on a high-performance computing (HPC) cluster ~~the server~~ of the Center for Research Computing (CRC), University of Notre Dame, IN, USA. For the pilot study, raw sequencing data was transferred from ZAMEP to the HPC cluster but was slowed down by a slow internet connection.”

17) Ensure consistency: In line 230 “version 6.1.7” is stated but in line 215 “v22.05.8” is used. I would change “v” to “version” and keep consistent throughout.

**RESPONSE:** We have changed “v” to “version” throughout the manuscript.

18) Line 670: Would be great to specify other disease control and elimination programs. Give an example. You could survey soil-transmitted helminths for example as well as malaria should the capacity be built in country.

**RESPONSE:** We have slightly changed the sentence to make it clearer that this likely also applies to malaria control programs of other countries in Africa as well as to control/elimination programs targeting other diseases, e.g., soil-transmitted helminths (see page 28, lines 703-706).

“These challenges must be addressed in the frame of establishing a robust in-country malaria sequencing program in Zanzibar and other African countries ~~most likely other disease control and elimination programs in Africa~~. Furthermore, these challenges are likely to apply to control and elimination programs targeting not only malaria but other pathogens (e.g., soil-transmitted helminths) as part of broader pathogen genomics initiatives (17, 61).”

19) Line 621: you could add my comments earlier about investment in a high-performance desktop computer instead of focusing on a laptop. If one of these desktop computers were used, you would actually decrease computing time compared to the MinION device. “Setting up a high range desktop computer in the ZAMEP laboratory and using hard drives to transfer data would be the best option. For example, you could get an Intel Core-i9 13900KS processor, RTX4090 graphics, 128GB DDR5 RAM and over 25TB of storage for around $5000-$8000.”

**RESPONSE:** We have added this (see response to comment 12).

Great study!

**RESPONSE:** Thank you very much!

We note that in Figure 4 and Supplementary Figure S4, there was an error in the Figure legend. The ratio used was 70:30 and not 80:20. We have corrected this minor typo throughout.
